# Impact of mass drug administration for elimination of lymphatic filariasis in Nepal

**DOI:** 10.1371/journal.pntd.0005788

**Published:** 2017-07-19

**Authors:** Chet Raj Ojha, Basant Joshi, Khagendra Prakash KC, Shyam Prakash Dumre, Keshav Kumar Yogi, Bandana Bhatta, Tulasi Adhikari, Kathryn Crowley, Babu Ram Marasini

**Affiliations:** 1 Department of Immunology, Herbert Wertheim College of Medicine, Florida International University, Miami, Florida, United States of America; 2 Leadership for Environment and Development Nepal, Kathmandu, Nepal; 3 Department of Immunogenetics, Institute of Tropical Medicine, Nagasaki University, Nagasaki, Japan; 4 World Health Organization, Lalitpur, Nepal; 5 Save the Children International, Kathmandu, Nepal; 6 Epidemiology and Disease Control Division, Ministry of Health and Population, Government of Nepal, Kathmandu, Nepal; 7 RTI International, Washington, District of Columbia, United States of America; RTI International, UNITED STATES

## Abstract

**Background:**

Lymphatic filariasis (LF) is a neglected tropical disease transmitted by mosquitoes. Nepal has implemented a national effort to eliminate LF by 2020 through mass drug administration (MDA) using diethylcarbamazine (DEC) and albendazole (ALB). We assessed the impact of MDAs on LF in selected districts of Nepal after the recommended six MDA rounds had been completed.

**Methodology and principal findings:**

Baseline surveys were conducted in seven districts and mapping data were used as baseline in the other three districts before starting MDA in 2009. LF antigen (Ag) prevalence ranged from 1.06% to 20% among districts included in the baseline and mapping study. The number of people who received DEC and ALB were recorded during each MDA round and population-based cluster surveys were conducted at least once in each district during the life of the program. The reported MDA coverage in five districts was consistently at least 65%. Two districts achieved the targeted coverage in four out of five rounds and the rest three districts achieved the target only in the first round. A pre-transmission assessment survey (pre-TAS) was conducted in one sentinel site and at least one spot check site in each of the districts after five MDA rounds. In pre-TAS, all the sites of five districts (Pyuthan, Arghakhanchi, Kaski, Bhaktapur, and Kathmandu) and all but one spot check site of Lalitpur district had LF Ag < 2% (ranging from 0.0% to 1.99%). Transmission assessment survey (TAS) was conducted in six evaluation units (EUs) consisting of six districts qualified on pre-TAS. Though MDA coverage of 65% was not achieved in three districts (Kathmandu, Lalitpur and Bhaktapur), Nepal government in consultation with World Health Organization (WHO) decided to conduct TAS. All six EUs achieved the LF Ag threshold required to stop MDA in TAS, despite the low reported MDA coverage in those three districts.

**Conclusions:**

Although Nepal has achieved significant progress towards LF elimination, five rounds of MDA were not sufficient to disrupt the transmission cycle in all districts, probably because of high baseline prevalence.

## Author summary

Lymphatic filariasis (LF) is a deforming and disabling infectious disease manifested in the form of elephantiasis and hydroceles. Based on mapping data, 61 out of 75 districts of Nepal were found to be endemic for LF and mass drug administration (MDA) of diethylcarbamazine and albendazole has been already started in 56 districts. The objective of MDA is to interrupt transmission of *Wuchereria bancrofti* in LF endemic districts. In 2015, ten districts had completed six MDA rounds. In this study we reported the impact of the national LF elimination effort in these ten districts of Nepal. The reported coverage rate in each MDA round was consistently above 65% in five districts. Compared to the baseline surveys, pre-transmission assessment surveys (pre-TAS) conducted after completion of five MDA rounds showed reductions in LF antigenemia (Ag) in most districts. Six out of ten districts (formed into six evaluation units) were eligible for TAS, which showed LF Ag below the cutoff point in all six EUs, suggesting successful interruption of LF transmission in six out of ten districts after six MDA rounds.

## Introduction

Lymphatic filariasis (LF) is a vector-borne neglected tropical disease of human caused by *Brugia malayi*, *Brugia timori* and *Wuchereria bancrofti*, and transmitted by *Culex*, *Anopheles* and *Aedes* spp. mosquitoes [1]. Infection with filarial worms can cause significant morbidity (primarily lymphedema of legs, arms and breast, and hydrocele) and disability, impeding socioeconomic development in many endemic countries [2,3]. Globally, 120 million people are estimated to be affected by LF and 40 million suffer from chronic disability and covert lymphatic changes caused by LF [4]. The Global Programme to Eliminate Lymphatic Filariasis (GPELF) was established by the World Health Organization (WHO) in 2000 with the aim to eliminate LF by 2020. To achieve GPELF targets, endemic countries conduct annual mass drug administration (MDA) of the population at risk of LF using diethylcarbamazine (DEC) and albendazole (ALB) to interrupt the transmission of filarial worms, along with the management of the disease’s chronic manifestations [5,6]. Although, ivermectin has shown to be more effective in killing filarial worms, Nepal uses DEC because it is more readily available and has less adverse effects than ivermectin [7,8].

In Nepal, *W*. *bancrofti* is the filarial worm that causes LF and *Culex quinquefasciatus* is the main vector [9]. Of Nepal’s 75 districts, 61 are considered LF endemic, corresponding to an estimated 25 million people at risk of infection and disease. Nine districts have historical evidence of chronic cases of LF. Fifty-two we classified as endemic following mapping of 54 districts between 2001 and 2005 using immunochromatographic tests (ICTs) to test for antigenemia (Ag) and using night blood films to test for microfilaremia (mf). Twelve districts are not suspected to be LF endemic and were not mapped. Mapping surveys indicated that prevalence in endemic districts’ surveyed villages ranged from <1% to 39% [10,11].

In 2013, the Government of Nepal developed a Plan of Action to eliminate LF by 2020 via implementation of six MDA rounds in all endemic districts until 2018 [12]. Nepal started LF MDAs in Parsa district in 2003 and achieved 100% geographical coverage of all districts in 2013. By 2016, 16 out of the 61 endemic districts had conducted six MDA rounds [13]. Effectiveness of MDAs at reaching the target population, as measured by epidemiological coverage, ranged from 22% to 89%, with differences noted both among districts and years. Reasons for low MDA coverage included fear of side-effects, lack of advice from health workers, and fear of weakness [12].

In addition to reporting drug coverage information following each round of MDA, WHO-recommended population based cluster surveys are conducted to verify reported MDA coverage data. To date, the program has conducted at least one coverage survey in each of the endemic districts that have had an MDA. WHO LF elimination guidance recommends that after 5–6 MDA rounds impact of MDAs on district Ag prevalence is assessed using a pre-transmission assessment survey (pre-TAS) in sentinel and spot-check sites. Furthermore, districts in which sentinel and spot check sites passed a cut-off LF Ag <2% and exceeded 65% coverage in all MDA rounds are considered to meet criteria for conducting a transmission assessment survey (TAS) to evaluate that LF transmission has been interrupted [4,12,14]. TAS is carried out in school children within an eligible district’s evaluation unit (EU) in order to determine interruption of LF transmission [14]. The objective of the study reported here was to assess the effectiveness and impact of MDAs in ten representative districts of Nepal in line with the country’s ongoing efforts to eliminate LF.

## Methodology

### Ethical statement

Ethical approval for the study was obtained from the Nepal Health Research Council. A written consent was taken from all survey participants before subjecting to diagnostic testing. In case of minors, parental consent was obtained. People with positive ICT and Filariasis Test Strip (FTS) results were referred to the government health facility (Health Post, Primary Health Center) for appropriate management as per national LF guidelines.

### Baseline survey

Between 2005 and 2008, the Government of Nepal conducted baseline surveys in seven of ten purposively selected districts (Lalitpur, Parbat, Myagdi, Baglung, Arghakhanchi, Pyuthan, and Lamjung), before starting MDAs. For each district, two sentinel sites, each with populations between 300 and 500 people, were selected; population size, migration rate and estimated LF prevalence were factors considered when selecting sentinel sites. In three districts, Bhaktapur, Kathmandu, and Kaski, no baseline surveys were conducted; instead the National LF Program used mapping data, collected in 2001 from one site per district, to determine that each district met the MDA eligibility criteria.

### MDA coverage data

During each MDA round drug distributors recorded the number of people who received DEC and ALB, and this data was communicated through the National LF Program’s reporting system. Recorded data were then reported to the Ministry of Health and Population (MOHP) and to WHO’s Preventive Chemotherapy (PCT) databank. The GPELF recommends that national programs should try to attain an epidemiological drug coverage of 65% when conducting MDAs for LF [14]. In order to verify reported drug coverage rates and determine reasons for non-compliance, the National LF Program conducted population-based cluster surveys at least once in each district during the life time of MDAs. For each of these surveys, a two-stage sampling approach was used, with 30 clusters randomly sampled per district and 30 households randomly sampled per cluster. All household members available at the house during the visit were interviewed; in the case of household members <10 years of age, the information was collected from their primary caretaker.

### Pre-transmission assessment survey

**Fig 1:**
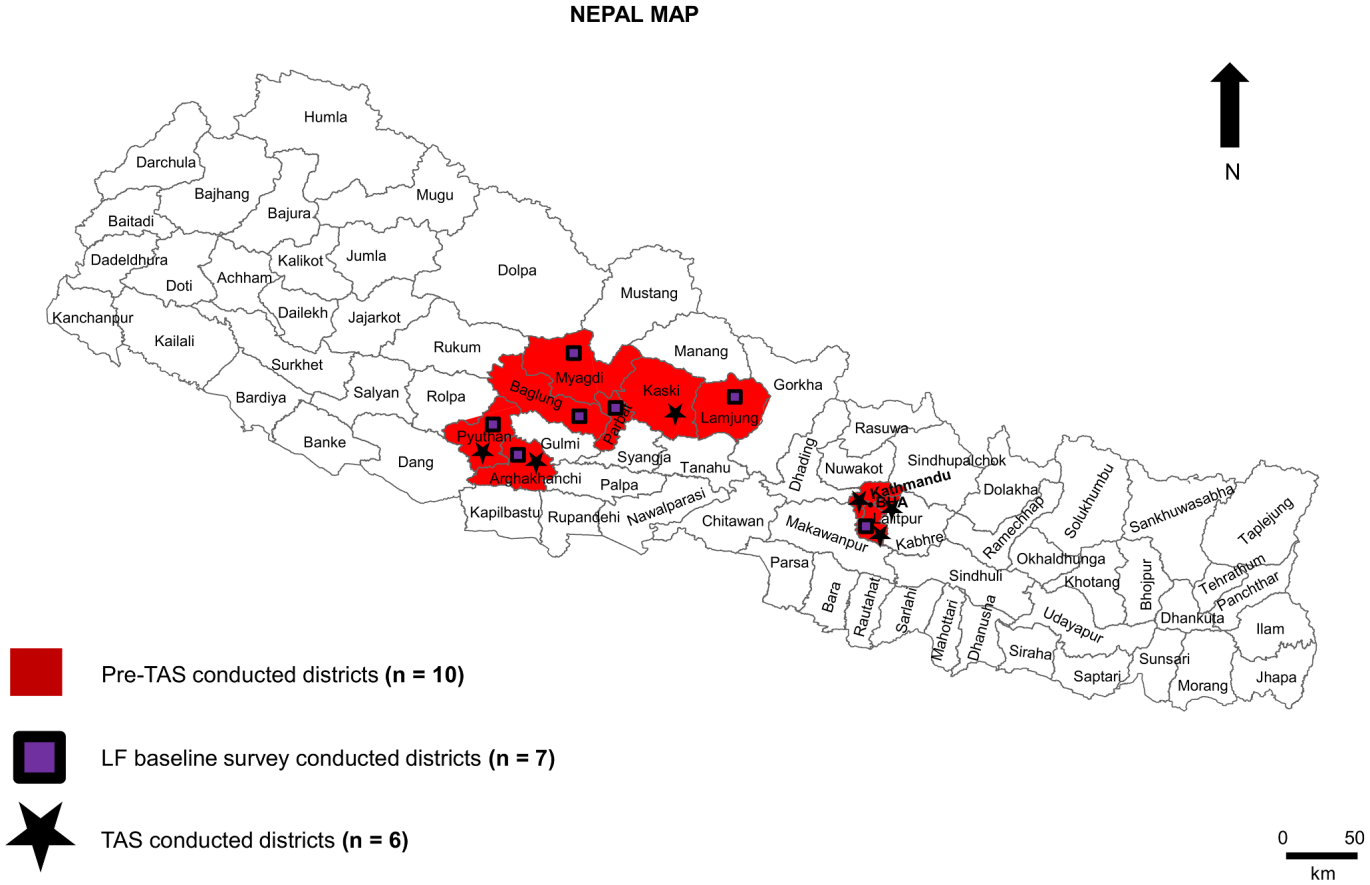
Map of Nepal showing the districts where the studies were conducted. Baseline survey was conducted in seven districts indicated by blue square, Pre-Transmission Assessment Survey was conducted in 10 districts indicated by red color and Transmission Assessment Survey was conducted in six districts indicated by star symbol.

A pre-TAS was conducted in each of the ten districts (Fig 1) after completion of five MDA rounds. Within each district, one sentinel site (same site where baseline or mapping survey was conducted) and one spot check site was selected for the study. Owing to the high population density and low (<65%) coverage of MDA in Kathmandu, Lalitpur, and Bhaktapur, additional spot check sites were purposively selected to assess LF transmission in those urban populations; sentinel site selection criteria were the same as for the baseline surveys. All people of more than 5 years of age from selected sites were included in the study. Wherever the population was too large (more than 500), systematic random sampling was used to select a representative sample. A total of 9,000 people (300 from each site) were tested for LF Ag. In addition, qualitative and quantitative data were collected using a structured questionnaire to assess MDA compliance. Information collected included whether the person took DEC and ALB during the MDA, experience of side-effects of the drugs, and signs and symptoms of LF disease.

### Transmission assessment survey

A TAS was conducted in six districts (Kathmandu, Lalitpur, Kaski, Pyuthan, Arghakhanchi and Bhaktapur) that had met TAS eligibility criteria (Fig 1). District evaluation units (EUs) were determined based on WHO TAS guidance, taking into account population size, baseline and mapping prevalence, and other LF risk factors [14]. The net primary school enrollment was >75% in all districts to be surveyed, so TAS were conducted in schools within each EU. The Ministry of Education provided lists of enrolled students in all schools—public, private, and religious—within the district. The sample size, critical cut-off value and school sampling frame in each EU were determined by systemic random sampling using the Survey Sampler Builder Software (SSB, NTD Support Center, Task Force for Global Health, Atlanta, GA). The SSB generated the required sample size for each EU, list of schools to be sampled within the EU with systemic intervals, and critical cut-off values. All children studying in grade one and/or two of selected schools were included for survey, with a goal of sampling children aged 6–7 years of age. If children were not in school on the day of the survey, they were not included in the TAS. From each EU, ≥1,540 school children were tested for LF Ag. The critical cut-off value for all EUs to pass TAS—as generated by the SSB—was 18 children testing positive for LF Ag.

### Circulating filarial antigen screening test

The Binax Now Filariasis ICT (Alere Inc., Scarborough, ME) was used to detect the filarial antigen circulating in the blood stream in the baseline and pre-TAS studies. Briefly, capillary blood was collected from survey participants by finger prick using special sterile lancets and lancet holders. A graduated capillary tube having a capacity to hold 100 μl of blood was used to take blood from participants and blood was dispensed onto the test card immediately. For TAS, the Alere Filariasis Test Strip (FTS, Alere Inc., Scarborough, ME) was used. In this case, 75 μl of finger pricked whole blood was collected from the survey participants and applied to the test strip by capillary tube. The test results were interpreted strictly following the manufacturer’s instructions. Positive control filarial antigen was used to confirm the quality of the ICT and FTS as per provided instructions.

### Statistical analysis

All the data, including test results, were entered into Microsoft Excel (Microsoft Corporation, Redmont, WA). Descriptive statistics were used to describe the basic features of the data in the study; the prevalence of LF Ag was determined by calculating the percentage of positive cases out of the total population examined for pre-TAS and TAS assessments.

## Results

### LF program coverage

Among the ten districts included in the study, only five (Arghakhanchi, Pyuthan, Kaski, Baglung, and Myagdi) consistently achieved at least 65% epidemiological coverage (Table 1). Lamjung and Parbat each achieved the threshold in four MDA rounds, with only the MDA in 2012 falling short of the 65% coverage. The other three districts, Kathmandu, Lalitpur, and Bhaktapur, are three heavily urban and peri-urban districts that make up the Kathmandu Valley. While reported epidemiological coverage exceeded 65% in all three districts for their first year of MDA, reported coverage declined markedly in 2011 (Bhaktapur, 43.1%) and 2012 (Kathmandu, 48.2% and Lalitpur, 40.1%), staying <65% in subsequent MDA rounds.

**Table 1:** Reported MDA coverage and surveyed coverage, 2010–2015.

District	Population	Reported MDA coverage (%)						Surveyed coverage (%)	
		2010	2011	2012	2013	2014	2015	Year (s)	Results
Kathmandu	1,916,667	74.0	69.6	*48*.*2*	*44*.*0*	*42*.*0*	*45*.*7*	2013 2015	*36*.*6* *41*.*8*
Lalitpur	505,490	68.3	67.8	*40*.*1*	*45*.*2*	*57*.*9*	*58*.*1*	2014	*34*.*3*
Bhaktapur	327,907	68.3	*43*.*1*	*45*.*9*	*44*.*6*	*46*.*7*	*44*.*5*	2014	*22*.*9*
Kaski	527,439	75.2	82.8	78.1	71.3	73.3	74.5	2014 2015	*59*.*6* *58*.*3*
Arghakhanchi	198,559	72.5	74.1	65.7	81.1	83.2	79.1	2014	87.7
Pyuthan	231,756	69.0	79.1	68.7	85.8	83.1	84.6	2015	89.4
Lamjung	168,652	75.4	78.8	*62*.*0*	81.6	81.1	77.8	2015	70.5
Baglung	273,614	80.1	71.5	66.7	69.3	80.8	80.1	2015	83.6
Myagdi	112,439	82.4	78.9	75.1	88.7	84.2	82.9	2014	87.8
Parbat	146,962	73.6	70.1	*63*.*4*	81.8	78.9	83.3	2015	78.4

In italics, coverage not meeting the 65% epidemiological coverage threshold

Results from coverage surveys validated reported district MDA coverage exceeding (Arghakhanchi, Myagdi, Pyuthan, Lamjung, Baglung, and Parbat) or missing (Kathmandu, Lalitpur, and Bhaktapur) the 65% threshold. Results from Kaski in 2014 and 2015 indicated that—although epidemiological coverage had reportedly met the 65% threshold—surveyed coverage was less than 65%. Respondents noted fear of side-effects, illness, and absence as the main reasons for noncompliance.

### Pre-TAS and TAS results

#### Pre-TAS results

In five out of ten districts (Pyuthan, Arghakhanchi, Kaski, Bhaktapur, and Kathmandu) all sentinel and spot check sites reported results of <2% LF Ag. In Lalitpur, all but one spot check site (3.0%), also had <2% LF Ag. In these six districts, there was a marked decrease between baseline and pre-TAS surveys. In Kathmandu and Bhaktapur, initial mapping in 2001 indicated a LF Ag of 20.0% and 19.8%, respectively. Despite poor reported and surveyed MDA coverage, Kathmandu and Bhaktapur pre-TAS results ranged from 0.33% to 0.67% and 0.0% to 0.33% respectively. In Kaski, LF Ag decreased from 7.3% reported in the 2001 mapping survey to 1.29% in the 2014 pre-TAS. Both Arghakhanchi and Pyuthan had relatively low prevalence at baseline in 2005 (1.9% and 2.2%, respectively). Arghakhanchi appears to have similar pre-TAS results to baseline (1.99% at site with highest number of positives), while Pyuthan had zero positives at each of the survey sites. In all but one of the sites from Lalitpur, LF Ag ranged from 0.0% to 0.98%, which is below the 2.0% LF Ag threshold; one spot check site in a peri-urban area of Lalitpur reported a LF Ag of 3.0% (Table 2).

**Table 2:** Summary of results from baseline and pre-TAS.

District	Population	Baseline		Pre-TAS					
		Year	Infection prevalence (%)	# sentinel sites	# persons tested per site	LF Ag (%)	# spot check sites	# persons tested per site	LF Ag (%)
Kathmandu	1,916,667	2001	20.0^	1	300	0.3	5	300	Range: 0.33–0.67
Lalitpur	505,490	2008	1.06*	1	300	0.66	5	300	Range: 0.0–3.0
Bhaktapur	327,907	2001	19.8^	1	300	0.0	3	300	Range: 0.33–0.67
Kaski	527,439	2001	7.3^	1	300	0.97	1	300	1.28
Arghakhanchi	198,559	2005	1.9^	1	300	1.99	1	300	0.98
Pyuthan	231,756	2005	2.2^	1	300	0.0	1	300	0.00
Lamjung	168,652	2008	5.76*	1	300	5.66	1	300	0.0
Baglung	273,614	2005	12.4^	1	300	3.0	1	300	0.0
Myagdi	112,439	2005	11.8^	1	300	0.66	1	300	2.3
Parbat	146,962	2008	4.83*	1	300	0.97	1	300	2.5

^, ICT used at mapping;

*, blood film (Mf) used at mapping

Four districts had LF Ag of ≥2% and, thus, failed to meet the TAS eligibility criteria. Baglung and Myagdi both reported a baseline LF Ag exceeding 10.0% in 2005 and experienced considerable decreases in LF Ag at sentinel and spot-check sites, but did not meet the 2% TAS threshold (Baglung’s sentinel site: 3.0%; Myagdi’s spot-check site: 2.3%). In the other two districts, Parbat and Lamjung, with mapping mf prevalence 4.83% and 5.76% respectively, were also not qualified for TAS (Table 2).

Out of total number of people tested during pre-TAS in all districts, only five (0.06%) reported being affected with chronic symptoms of LF. Out of those five cases, four were reported from Bhaktapur and one from Myagdi. Interestingly, the case in Myagdi was a child under the age of 9 years with positive Ag.

We asked pre-TAS participants if they had taken drugs during at least one out of five MDA rounds. The MDA compliance rate reported during pre-TAS was slightly higher than the reported MDA coverage in all ten districts (Table 3). In Lalitpur, Bhaktapur, Kaski, Arghakhanchi, and Lamjung, the LF Ag was higher among participants who had never taken the drugs during MDA.

**Table 3:** LF antigenemia in pre-TAS survey based on MDA participation.

District	Compliance rate (%)	Number of Non compliants examined for Ag	Ag among non- compliants	Number of compliants examined for Ag	Ag among compliants
Kathmandu	59	730	0.4	1,070	0.7
Lalitpur	65	595	1.8	1,205	0.3
Bhaktapur	49	606	0.2	594	0.0
Kaski	89	60	1.6	540	1.1
Arghakhanchi	90	53	1.7	547	1.5
Pyuthan	90	56	0.0	544	0.0
Lamjung	92	47	6.4	553	2.5
Baglung	93	45	0.0	555	1.6
Myagdi	91	50	0.0	550	1.6
Parbat	90	55	1.8	545	1.8

#### TAS results

Based on the pre-TAS results, six districts, grouped into six EUs, went on to conduct a TAS. Arghakhanchi did have one site with LF Ag of 1.99%, but following consultation with WHO, it was decided that this was still sufficient for meeting TAS eligibility. Kathmandu, Lalitpur, and Bhaktapur were of concern to the National LF Program. While all sentinel and nearly all spot check sites had LF Ag <2% at pre-TAS, these districts failed to attain epidemiological coverage of ≥65% for all five MDA rounds and surveyed coverage was even lower than reported MDA coverage. As a result, the Ministry of Health and Population (MOHP) consulted with WHO staff at a SEARO meeting in Puducherry, India, in June 2015. Based on the LF Ag data collected and the concern that these districts would—if continuing to implement MDAs—never reach the required epidemiological coverage, WHO advised the MOHP to proceed with TAS. For Lalitpur, given that one spot check site reported a LF Ag >2%, WHO advised splitting the district into two EUs. The National LF Program divided Lalitpur into “rural” and “urban” sub-districts, with the “urban” sub-district advancing to TAS, and the “rural” sub-district including the peri-urban spot check site with 3% LF Ag underwent additional two MDA rounds in 2015 and 2016. Since Kathmandu’s population was 1.9 million, the National LF Program decided to divide the district into two EUs. Arghakhanchi and Pyuthan were grouped together into one EU, given their similar baseline prevalence and geographic proximity. TAS implementation in six EUs showed that all six achieved the prevalence threshold required to stop MDA (Table 4).

**Table 4:** Summary of results from TAS.

Name of evaluation unit	Name of IU	Diagnostic test	Age group surveyed in years (Min-Max)	Target sample size	Number of people examined	Number of people positive	Number of people for critical cut off	Pass/Fail
KTM-Rural	Kathmandu	FTS	6–9	1,556	1,562	4	18	Pass
KTM- Urban	Kathmandu	FTS	6–9	1,556	1,587	8	18	Pass
LAL-Urban	Lalitpur (partial)	FTS	6–9	1,540	1,600	6	18	Pass
Bhaktapur	Bhaktapur	FTS	6–9	1,540	1,579	2	18	Pass
Kaski	Kaski	FTS	6–9	1,556	1,566	2	18	Pass
Argha-Pyuthan	Arghakhanchi	FTS	6–9	1556	1601	0	18	Pass
	Pyuthan							

## Discussion and conclusion

The impact of MDA to eliminate LF transmission is determined by the reduction of LF Ag below a critical cut-off in each EU as measured by the TAS [14]. In the current study, LF prevalence was shown to be reduced in ten districts that underwent at least five MDA rounds with DEC and ALB. Six out of ten districts showed decreased LF Ag in pre-TAS compared to baseline. In five of the six districts, results indicate that transmission was interrupted; part of a sixth district, Lalitpur, was also found to have interrupted transmission.

Though the TAS sampling method was different to the one used for the baseline survey and pre-TAS, continuous reductions in LF Ag were observed from the first survey done in 2001 to surveys done in 2015. In a study conducted by Sherchand et al. [11] in 2001, LF Ag was reported to be 20% from Kathmandu, 19.8% from Bhaktapur, and 7.3% from Kaski among the adult population (>15 years of age). Substantial decreases in LF Ag were observed at pre-TAS compared to baseline surveys, the exception being a spot check site in Lalitpur. In nine out of ten districts no children below 9 years of age were positive for LF Ag. Although LF Ag had been reduced in all districts, only six districts passed the eligibility criteria to advance to TAS. In the other four districts (Parbat, Lamjung, Myagdi and Baglung) five rounds of LF MDA with consistently good coverage had an impact on transmission, but not sufficient to interrupt LF transmission completely—it is expected that additional rounds of MDA in these districts are required to do so. Districts/EUs that conducted TAS showed LF Ag below the established cutoff, and MDAs in those district can be discontinued.

The Nepal National LF Program faced a significant challenge as a result of Serious adverse effects (SAEs) that occurred during MDAs in 2011. SAEs included several deaths, and were widely publicized in Nepal. An investigation was conducted by the MOHP and the WHO, which determined that the deaths were coincidental and not related to taking the drugs; quality testing of the DEC and ALB was done to ensure they met national quality standards. Moreover, the national program provided additional education to drug distributors about the recommended drug dosages (6 mg/kg DEC and 400 mg ALB). Drug distributors and the population received clearer information about potential drug side effects and what to do if adverse or SAEs were experienced. People who experienced nausea, fever, headache, dizziness, or other symptoms after taking the drugs were encouraged to visit the nearest health facility immediately and the National LF Program provided management, as required. A policy was also established for drugs to only be distributed in the winter months, to avoid people from having to take them when working outside during hot summer months and possibly becoming dehydrated. Nonetheless MDA epidemiological coverage was affected, with nine out of ten districts experiencing a reduction in coverage in 2012, and the three largely urban and peri-urban districts of Kathmandu, Lalitpur, and Bhaktapur never exceeding 65% coverage in subsequent MDAs.

The impact of MDA on LF transmission in Nepal was found to be considerable and in line with findings observed in India, Papua New Guinea, and Egypt [15–17], all of which conducted MDA with DEC and ALB. In a couple of LF endemic districts of India, the LF mf and LF Ag prevalence was 0.2% and 2.3%, respectively, after eight MDA rounds [17]. In a multicenter study carried out in different countries, 10 out of 11 countries showed LF prevalence below the 2% cutoff point after completion of five MDA rounds [18]. Compliance is one of the major factors that influence the interruption of LF transmission. Studies have reported higher LF Ag after several MDA rounds among non-compliant than compliant population groups [19,20]. Poor compliance has been reported from India, where out of 99% of study participants who received DEC and ALB tablets during MDA, only 30% actually consumed the drugs [21]. Various factors such as lower educational status, higher age, lack of awareness and/or knowledge of LF and MDA, unwillingness of health care workers to visit for MDA distribution, and particularly unawareness of fear of side-effects are reasons for low MDA compliance [22–24]. Several studies from India have reported that non-compliance compromises MDA effectiveness [21,25–28]. Similarly, data from Egypt, where compliance was reportedly excellent (>85%) showed that the people who had never taken DEC and ALB had higher levels of mf and LF Ag in comparison to those who took all five MDA doses [16]. In our study, compliance as recorded during pre-TAS ranged from 49% to 93%, which in most of the districts (7 out of 10) was sufficiently higher than the expected >65% target. Reported MDA coverage rates ranged from 44–89%, which were validated by coverage surveys in nine of the ten districts included in this study.

In our study, the MDA coverage varied among the EUs which met TAS eligibility criteria based on pre-TAS data. Despite low MDA coverage and compliance in three districts (Kathmandu, Lalitpur and Bhaktapur), all six EUs passed TAS. This indicates that MDA coverage of around 50% may be sufficient to interrupt LF transmission in urban populations. In contrast, four rural districts having high MDA coverage were unable to stop LF transmission after five MDA rounds; we noted that these districts’ baseline LF prevalence was comparatively higher. Thus, MDA coverage is not the single factor that may determine the number of MDA rounds required to interrupt LF transmission, and other factors such as baseline LF prevalence, population density [29], occupation [30], personal hygiene [31], level of education and nutritional status [2] should be considered. Overall, our study showed the effective implementation of LF elimination efforts in Nepal, with some districts requiring additional MDA rounds to achieve LF transmission interruption.
